# A Novel Sensorized Heart Valve Prosthesis: Preliminary In Vitro Evaluation

**DOI:** 10.3390/s18113905

**Published:** 2018-11-13

**Authors:** Emanuela Marcelli, Barbara Bortolani, Ivan Corazza, Laura Cercenelli

**Affiliations:** 1 Laboratory of Bioengineering, DIMES Department, University of Bologna, S. Orsola-Malpighi Hospital, 40138 Bologna, Italy; emanuela.marcelli@unibo.it (E.M.); barbara.bortolani@unibo.it (B.B.); 2 Medical Physics Activities Coordination Center, DIMES Department, University of Bologna, 40138 Bologna, Italy; ivan.corazza@unibo.it

**Keywords:** heart valve prosthesis, valve thrombosis, electric impedance, implantable sensor, continuous monitoring

## Abstract

Background: Recent studies have shown that subclinical valve thrombosis in heart valve prosthesis (HVP) can be responsible for reduced leaflet motion detectable only by advanced imaging diagnostics. We conceived a novel sensorized HVP able to detect earlier any thrombus formation that may alter the leaflets motion using an electric impedance measurement, IntraValvular Impedance (IVI). Methods: For IVI measurement, dedicated electrodes are embedded in the structure of the HVP to generate a local electric field that is altered by the moving valve leaflets during their cyclic opening/closing. We present preliminary in vitro results using a first prototype of sensorized mechanical heart valve connected to an external impedance measurement system. The prototype was tested on a circulatory mock loop system and the IVI signals were recorded during both normal dynamics and experimentally induced altered working of the leaflets. Results: Recordings showed a very repetitive and stable IVI signal during the normal cyclic opening/closing of the HVP. The induced alterations in leaflet motion were reflected in the IVI signal. Conclusions: The novel sensorized HVP has great potential to give early warning of possible subclinical valve thrombosis altering the valve leaflet motion, and to help in tailoring the anticoagulation therapy.

## 1. Introduction

Valvular heart diseases affect more than 100 million persons worldwide and are associated with significant morbidity and mortality [1,2]. Surgical valve replacement of the diseased valve with a heart valve prosthesis (HVP) is currently the standard of care for patients at low and intermediate risk for surgery [3]. Moreover, in the last 10 years, there has been a proliferation of transcatheter aortic valve replacement (TAVR), alternately known as transcatheter aortic valve implantation (TAVI), which is a procedure in which a diseased aortic valve is replaced via an endovascular or transapical approach, using an expandable valve delivered with a catheter.

Based on the leaflet material, two different types of HVPs exist: Mechanical heart valves (MHVs) and biological heart valves (BHVs). MHVs are more thrombogenic, yet more durable. BHVs, made either of porcine origin or synthesized from a sheet of bovine pericardium, are less thrombogenic than MHV and exhibit more natural hemodynamic properties, but are less durable. All of the transcatheter HVPs consist of a porcine or bovine pericardial tissue mounted on a self-expandable or balloon-expandable metallic frame [3,4].

Despite continuing advances in surgical care and prosthetic design, complications after implantation of HVPs remain a substantial source of morbidity and mortality [5,6]. Indeed, all foreign bodies implanted within the cardiovascular system are thrombogenic, potentially implying the need for short- or long-term anticoagulation to prevent thrombosis, which can lead to a disabling or fatal stroke. HVP thrombosis, i.e., thrombus formation on the prosthetic structures, is a complication of mechanical or biological prostheses, which can cause reduced leaflet motion or impaired leaflet coaptation, reduced or increased effective prosthesis orifice area, increased transvalvular gradient, or transvalvular regurgitation, with or without development of valve-related symptoms [6,7,8].

Recently, many authors reported a reduced leaflet motion after BHVs implantation, more in transcatheter than in surgical valves, that might be associated with subclinical valve leaflet thrombosis and increased risk of stroke [9,10,11,12,13,14,15,16]. Of note, the reduced leaflet motion more commonly occurs in patients not receiving oral anticoagulants, and therapeutic anticoagulation is associated with resolution of reduced leaflet motion, supporting a thrombotic origin [9,17].

Reduced leaflet motion has been detected with the recent high-resolution 4-dimensional multi-detector computed tomography (4D-MDCT), that appears to be the most specific and sensitive imaging technique to provide an accurate evaluation of the prosthetic valve structure and functional status [9,18]. Moreover, there is evidence that the risk of BHV thrombosis can be not limited to within the first three months post-implantation, but it may persist beyond one year after valve implantation [19], which underscores the importance of serial imaging follow-up and a careful evaluation of the risks and benefits of long-term anticoagulation [13].

Given the exponential rise in TAVR procedures, it is important to detect the risk of subclinical valve thrombosis earlier to identify the optimal antithrombotic therapies/strategies. Additionally, in the MHVs, an early detection of reduced leaflet motion, due to thrombus formation often in conjunction with fibrotic pannus ingrowth, may be useful to reveal inadequate anticoagulant therapy for the patient.

Prevention and treatment of subclinical leaflet thrombosis might offer a potential opportunity for further improvement in valve hemodynamics and clinical outcomes. However, without routine imaging surveillance, this subclinical thrombosis may be underdiagnosed. Routine use of MDCT is not possible due to the potentially harmful effects for patients (radiation exposure and nephrotoxic effect of iodinated contrast media) and certain practical limitations related to significant logistical constraints in less-equipped centers and costs.

We have recently conceived an innovative sensorized HVP able to continuously monitor the valve leaflet motion after implantation [20,21,22]. The new sensorized HVP, which is based on an impedance measurement that we define IntraValvular Impedance (IVI), may have great potential for early detection of any possible subclinical valve leaflet thrombosis and progressive deterioration of the implanted prosthesis. We selected an impedance-based sensor over other possible sensing approaches like piezoelectric or magnetic type since impedance is a highly consolidated technique in the field of implantable cardiac devices [23,24], and according to our design it implies a less “intrusive” approach for the HVP since the valve leaflets can be maintained free from sensorization.

In this paper, we describe the conceptual design of this novel sensorized HVP based on IVI sensing, and the assembly of a first proof-of-concept prototype.

We also report in vitro results for the assembled prototype that was tested on a circulatory mock loop system when simulating both normal opening/closing dynamics and abnormal functioning of the valve leaflets.

## 2. Materials and Methods

### 2.1. IVI Sensor—The Concept

**Figure 1:**
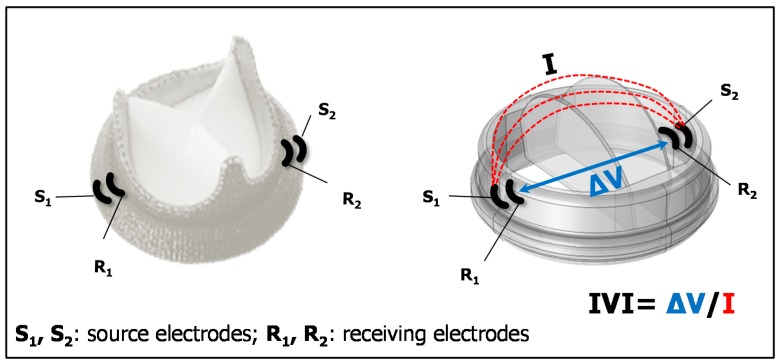
The principle of measurement of IntraValvular Impedance (IVI): Current pulses (I) are injected via source electrodes (S1, S2) to generate local electric field interfering with leaflet movement, and voltage (∆V) is recorded between receiving electrodes (R1, R2).

IVI measurement is based on the use of electrodes to generate a local electric field, by injecting sub-threshold currents (I), and to record electric field variations (V) caused by the moving leaflets of the valve prosthesis, which interfere with the local electric field lines (Figure 1). Following Ohm’s law, IVI measurement is defined as the recorded V over the injected I and its variations within the cardiac cycle (∆IVI) reflect the cyclic movement of the valve leaflets.

A preferred arrangement for the electrodes is tetrapolar configuration, i.e., the electric field generation is between a pair of source electrodes (S1, S2) and the voltage recording is between a different pair of receiving electrodes (R1, R2) (Figure 1).

Alterations of the optimal opening/closure dynamics of the valve leaflets can be detected as alterations of IVI signal.

### 2.2. The Proof-of-Concept Prototype

We sensorized a commercial mechanical mitral valve with two pairs of electrodes (S1–S2, R1–R2) that were connected by wires to an external impedance measurement system (Impact Custom Model 2364, Medtronic, Minneapolis, MA, USA) working with a low amplitude (36 μA) medium frequency (4 kHz) current signal.

**Figure 2:**
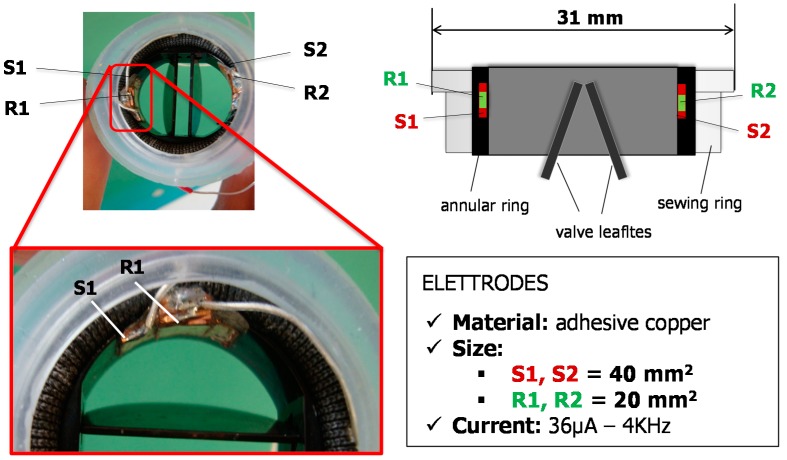
(**Left**) Photo of geometry and positioning of IVI sensing electrodes, as designed for the first proof-of-concept prototype of the novel sensorized HVP. (**Right**) Schematic representation of the cross-sectioned sensorized HVP. R1, R2: Receiving electrodes; S1, S2: Source electrodes.

We chose the inner wall of the valve ring for the electrodes positioning in order to concentrate the electric field lines in a region most sensitive to the interference caused by the moving leaflets. We designed an “overlapping” electrode geometry consisting of a square-shaped receiving (R) electrode (size 20 mm^2^) being surrounded by a source (S) electrode with double surface (40 mm^2^) (Figure 2).

The electrodes were made using adhesive conductive copper sheets that were carefully positioned in the valve ring to avoid the contact among the electrodes and the leaflets when the valve is in closed position. A substrate of insulator (double-sided adhesive plastic tape) was interposed between each electrode and the conductive surface of the valve ring, as well as between the R electrode and the surrounding S electrode, in order to prevent the contact between the two conductive copper layers (Figure 2).

### 2.3. In Vitro Tests

#### 2.3.1. In Vitro Test Bench

**Figure 3:**
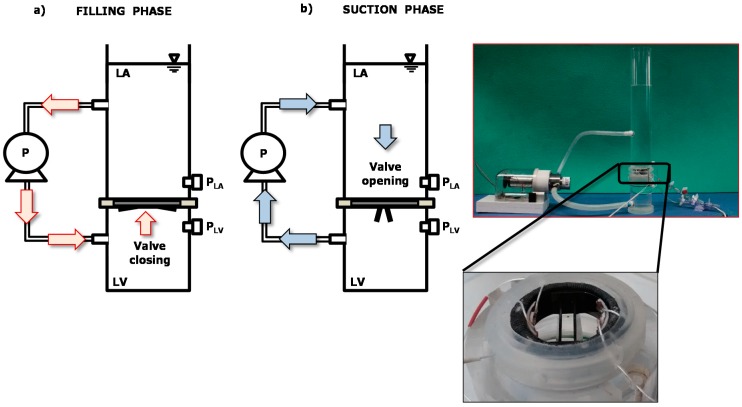
Scheme and picture of the mock loop system: (**a**) Filling phase that causes valve closing; (**b**) suction phase that causes valve opening. P = pump; LA = Left Atrium; LV = Left Ventricle; P_LA_ = LA pressure; P_LV_ = LV pressure.

The prototype was mounted on a simplified circulatory mock loop system that we assembled to reproduce hydrodynamic working conditions for the sensorized HVP. The system consisted of two cylindrical PVC chambers simulating, respectively, the left atrium (LA) and the left ventricle (LV), separated by a sealed silicone disc in which the sensorized mitral valve prosthesis was inserted (Figure 3). A gear pump (Maxon Motor AG, Sachseln, Switzerland), controlled by a motion control module (NI9505, cRIO, National Instruments, Austin, TX, USA), was used to regulate the flow rate and the direction of the circulatory fluid (saline solution) used in the mock loop. All the parameters for the system control can be set on a graphical user interface that we implemented in LabVIEW (National Instruments, Austin, TX, USA). When the pump fills the LV chamber, the pressure in this chamber increases and the mitral valve closes (early systole); when the pump sucks from the LV chamber the pressure decreases and the valve opens (diastole). Luer-lock connectors were provided for pressure measurement in the two chambers using standard pressure transducers (Baxter Uniflow, Bentley Laboratories Europe BV, Holland). LA pressure was maintained at about 10 mmHg by filling the LA chamber with 13 cm water column.

Different hemodynamic settings can be simulated by changing the flow rate (i.e., the gear pump velocity) and the duration of the filling and the suction phases.

#### 2.3.2. Tests for Selection of the Optimal IVI Measurement Configuration

Initial in vitro tests were conducted with normal functioning of the HVP, i.e., without any obstacle to the opening/closing of both leaflets. The mock loop system was programmed in order to simulate normal (HR = 60 bpm) cardiac times for valve closure (150 ms) and for valve opening (850 ms).

In this experimental condition, two different impedance measurement configurations were evaluated: The tetrapolar one, i.e., four distinct electrodes were used, two as sources (S1/S2) and other two as receivers (R1/R2), and bipolar one, i.e., the same electrode was used both as source and receiver (i.e., S1 ≡ R1, S2 ≡ R2). IVI signal was recorded for each configuration and IVI variations during the normal cyclic opening/closing of the valve were compared.

#### 2.3.3. Tests with Altered Leaflet Dynamics

**Figure 4:**
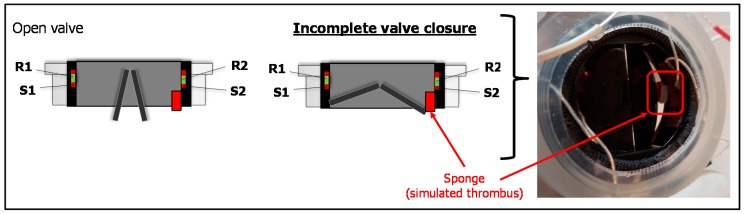
The experimentally reproduced alteration of valve leaflet dynamics: Incomplete valve closure due to the obstacle caused by the sponge simulating a thrombus formation.

A second set of tests were addressed to evaluate the IVI signal during altered functioning of the valve leaflets of the sensorized HVP that we experimentally reproduced. We used a small volume of sponge material (4 × 4 × 2 mm) and we attached it by a bi-adhesive tape to the upper edge of the valve ring in order to hinder the complete closure of the valve, thus resembling the effect of a thrombus formation (Figure 4). Then, we mounted the prototype on the mock loop and we performed the IVI measurement using the tetrapolar impedance configuration. The IVI recordings were then compared to IVI signals previously acquired for normal opening/closing dynamics, in the same tetrapolar impedance measurement configuration.

### 2.4. Data Analysis and Statistics

For comparative evaluation of the recorded IVI signals in the different experimental conditions, we considered the maximum percent variation of the impedance module (Δ*IVI_MAX_*%) when passing from a completely open position of the valve (*IVI_open_*) to closed one (*IVI_closed_*), as calculated in Equation (1).

(1) $$\Delta IVI_{MAX}\%=\frac{IVI_{open}-IVI_{closed}}{IVI_{closed}}\times 100$$

For each experimental condition, Δ*IVI_MAX_*% was reported as Mean value (±SD) calculated over 30 cardiac cycles, and for comparative evaluations the Student’s *t* test was used. All analyses were made with SPSS version 23.0 (IBM SPSS, New York, NY, USA) and a *p* value of 0.05 was chosen as significant.

## 3. Results

**Figure 5:**
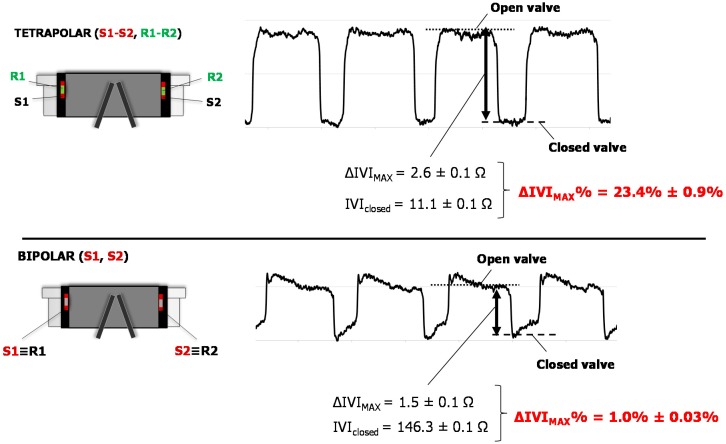
IVI signal recorded for tetrapolar (**top**) and bipolar (**bottom**) impedance measurement configurations.

In all tests, the recorded IVI signal showed a profile reflecting the opening/closing dynamics of valve leaflets. There was an increasing trend when both leaflets were opening to reach the maximum open position; when the leaflets started to close the signal decreased to reach the minimum when leaflets achieved the complete closure (Figure 5). The IVI signal was very repetitive, robust, and stable from one cycle to another.

### 3.1. Results of Tests for Selection of IVI Measurement Configuration

In Figure 5, IVI signals acquired both for the tetrapolar and the bipolar impedance configurations were reported (see also the Supplemental Video Materials Video S1_Tetra_normal, Video S2_Bipolar_normal).

Tetrapolar configuration showed the higher impedance variation passing from a completely open position of the valve to a closed one, when compared to bipolar one (Δ*IVI_MAX_*% = 23.4% ± 0.9% vs. 1.0% ± 0.03%, *p* < 0.05). This indicates that in tetrapolar configuration any small IVI variation during the valve opening/closing is more easily detectable than in bipolar configuration where the similar small variation should be detected on a very large base signal (Figure 5).

In bipolar configuration the IVI signal also showed a less stable pattern, especially in the “low” phase corresponding to “closed valve” (Figure 5, bottom). This may be due to the effects of electrode polarization impedances that exist at the interface between each electrode and the medium interface, which are generally quite high in cases of bipolar measurements.

### 3.2. Results of Tests with Altered Leaflet Dynamics

**Figure 6:**
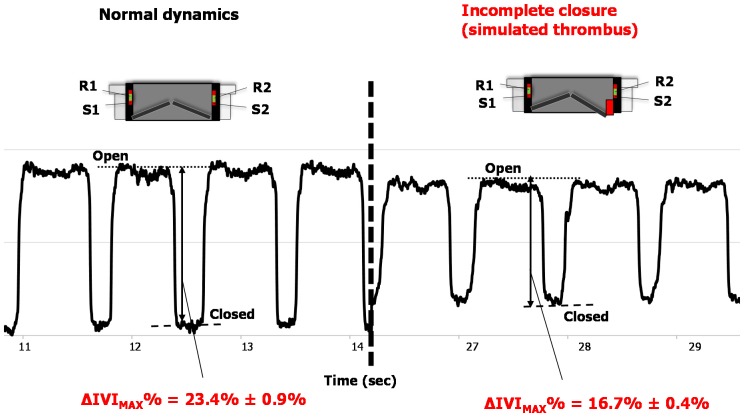
IVI signal recorded during normal opening/closing dynamics (**left**) and during the experimentally reproduced incomplete valve closure (**right**).

When reproducing the incomplete closure of the valve by interposing the sponge volume between one the two leaflets and the valve ring, the IVI signal showed a significant reduction of the overall variation between the valve in the completely open position and in the partially closed one (i.e., at the maximum closure allowed by the obstructing sponge), compared to normal leaflet dynamics (Δ*IVI_MAX_*% = 23.4% ± 0.9% vs. 16.7% ± 0.4%, *p* < 0.05). The acquired IVI signals for both the normal and the altered leaflet dynamics were shown in Figure 6 (see also the Supplemental Video Materials Video S1_Tetra_normal, Video S3_Tetra_altered).

## 4. Discussion

The described first prototype of sensorized HVP and the collected in vitro results are very innovative. To the authors’ knowledge, none of the current available HVPs, once implanted, is able to provide early detection of alarm situations that may alter the leaflet motion, such as subclinical thrombus formation, and that are hard or impossible to detect with standard clinical imaging techniques. If early detected, leaflet thrombosis could be efficiently treated with appropriate anticoagulant therapy with positive effect on patient safety and healthcare quality [9,17].

We think that IVI sensor is a very promising approach over other possible sensors, mainly for the following reasons. Differently from previously proposed sensing technologies, like the magnetic sensorization [25] that requires magnetic elements to be joined to the leaflets, the IVI sensor keeps the valve leaflets intact. A piezoelectric sensor (e.g., an accelerometer) surely could not be positioned on the valve leaflets but on the HVP annular structure in order to preserve the mechanical integrity and working condition of the leaflets [26]. Consequently, an accelerometer positioned in the annular ring could only detect vibrations resulting from the valve closure and not the overall valve leaflets dynamics as an impedance signal could provide since it reflects the interference of valve leaflet itself with the generated electric field lines. Moreover, electrical impedance or rheographic measurement in implantable cardiac devices (e.g., made between two or more electrodes inserted in a cardiac pacing lead) is a technique that has been used for more than thirty years and that is now consolidated in terms of current amplitude and frequency to be used, is easy to implement, and presents high technological reliability. So far, the rheographic technique has been used basically for detecting mainly parameters (like minute ventilation or atrioventricular impedance) for adapting pacing rate in an implantable electrical cardiac pacing device (rate responsive pacing) [23,24].

For our first prototype of sensorized MHV we chose to place the electrodes in the inner wall of the valve ring, and we accurately fixed them in a way to avoid any possible mechanical interference between them and the moving leaflets, both in the closing and the opening phase of the valve (see Figure 2). Considering this electrode position, we observed that when the valve was closed the IVI signal, measured in tetrapolar configuration, was low, while when the leaflets were at the maximum opening the IVI signal was high. This can be explained considering that when the valve is closed (i.e., leaflets are in horizontal position) the medium interposed between the IVI sensing electrodes is mainly the highly conductive pyrolytic carbon of which the leaflets are made. When the leaflets open, the medium between the electrodes changes into a mixture of pyrolytic carbon and blood (which is more resistive than pyrolytic carbon), therefore the IVI signal increases.

Our findings about greater sensitivity and reliability of tetrapolar impedance measurement configuration for IVI sensing were in accordance with the well-known concept that tetrapolar configuration allows to reduce the effects of interface impedances generated when the same electrodes are used as source and receiver (i.e., bipolar configuration) [27], despite the higher demanding issue of embedding more electrodes in the prosthetic valve.

### Study Limitations and Future Directions

The presented results are limited to in vitro tests of a wired first proof-of-concept prototype of sensorized MHV mounted on a circulatory mock loop that cannot reproduce the real in vivo environment when the sensorized HVP is implanted. This environment may have influence on the detected IVI signal. Therefore, future ex vivo experiments and in vivo animal experimentation are required to confirm these preliminary very interesting observations on IVI signal.

In this study, saline solution, which is less viscous than blood, was used as test fluid. However, in our tests the valve closing and opening times were regulated by the pumping system in order to maintain the physiological times occurring when fluid is blood. As further steps of our study, we are going to plan evaluations on ex vivo platforms using blood or blood-equivalent fluid as test fluid, as well as to get approval from in vivo experimentation on animals.

**Figure 7:**
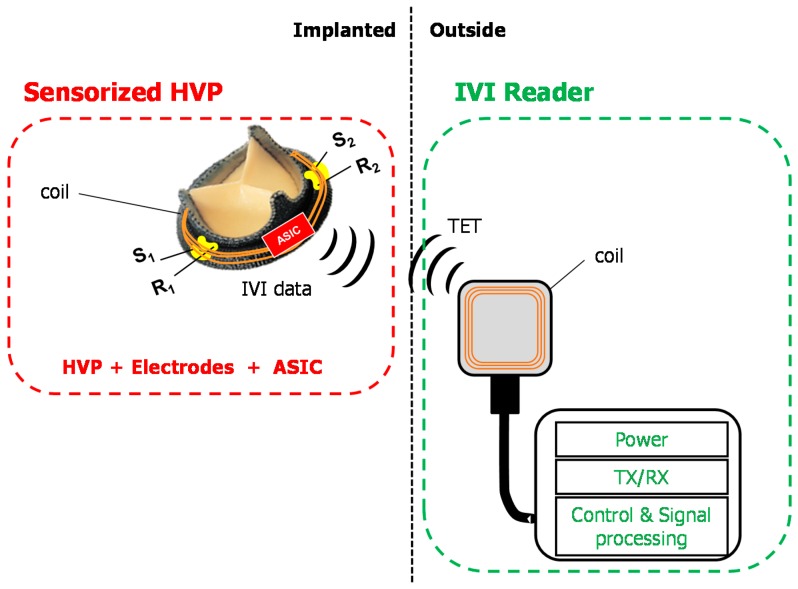
Scheme of the overall design for an implantable sensorized HVP for IVI sensing.

In the future, intensive work will be addressed to achieve an implantable prototype of the sensorized HVP for IVI sensing. In our design of the final implantable device, the sensorized HVP will be equipped with a miniaturized Application-Specific Integrated Circuit (ASIC) and means for telemetric communication with an external electronic unit (IVI reader) that wirelessly powers and interrogates the implanted sensorized system (Figure 7). The ASIC will mainly include a constant current pulse generator, an electrode connection unit, a signal conditioning (filtering/amplification) unit, and a TX-RX unit. Current injection will be tuned following the well-established ranges used in commercial impedance minute ventilation sensors for implantable cardiac devices [23,24] and the previous experience we collected in this field [28]. In detail, sub-threshold current pulses within the range of 200–400 µA amplitude (A), 10–100 µs pulse duration (D), and 100–200 Hz repetition rate (r) will be used.

Custom antennas will be also included in the implanted sensorized valve and the system will be powered by inductive coupling from the external IVI reader via transcutaneous energy transfer. The raw impedance data will be transferred to the reader by RF transmission for further processing of the IVI signal. By assuming to use typical current pulses of A = 300 μA, D = 15 μs, r = 128 Hz, and to interrogate the sensorized system every 10 s, we estimated a current consumption of about 1 mA. Efforts will be made to keep the overall power consumption of the novel implantable device as low as possible.

Future studies will be addressed to design a prototype of sensorized BHV and prosthesis that would greatly benefit from IVI sensing. Different solutions for the electrode positioning on the BHV structure (e.g., included in the suture ring or embedded in the supporting stent structure) will be studied and tested. Moreover, different biomaterials and manufacturing techniques will be evaluated for realizing the electrodes in the final implantable prototype in order to ensure their long-term biocompatibility and optimal embedding into the HVP structure.

In parallel to additional in vitro evaluations using a recently developed cardiac mock system with a good physiological behavior [29], we are working on the development of reliable in silico simulations reproducing the IVI measurements for different models of HVPs, different electrode size, geometry and positioning, and different working conditions for the valve leaflets. These additional studies and experiments will help to further improve the final design of our sensorized HVP.

Finally, we will work on algorithms for IVI signal processing, e.g., including the computation of additional IVI-derived parameters, such the first derivative of the recorded IVI signal that should be reflective of the velocity profiles of the moving leaflets, with further investigation of their possible correlation with blood flow rate across the prosthesis.

## 5. Conclusions

In this work, we demonstrated that the novel concept of sensorized HVP based on IVI sensing is of value and feasible for the continuous monitoring of the valve leaflet motion. From the collected in vitro results, IVI sensing seems to keep great potential for early warning of subclinical leaflet thrombosis, allowing the timely therapeutic intervention before the aggravation, thus improving patient quality of life, and healthcare efficiency and efficacy.

Additional efforts are required to achieve an implantable version of this innovative sensorized HVP and to evaluate it on in vivo animal experimentation.

## 6. Patents

From the work reported in this manuscript the following PCT patent applications result:

- WO2015EP58201 20150415. Heart valve prosthesis with integrated electronic circuit for measuring intravalvular electrical impedance, and system for monitoring functionality of the prosthesis. E. Marcelli (Inventor); Alma Mater Studiorum (Applicant). Filed: 15 April 2015.
- Also published as: EP3131502 (A1); CN106456043 (A); US9987129 (B2)—Issued: 5 June 2018.
- N. 0001423344 Protesi valvolare cardiaca con circuito elettronico integrato per effettuare misure di impedenza elettrica intravalvolare e sistema per monitorare la funzionalità di tale protesi—E. Marcelli (Inventor); Alma Mater Studiorum (Applicant). Filed: 16 April 2014. Issued: 22 July 2016.

## Supplementary Materials

The following supplementary video materials are available online at http://www.mdpi.com/1424-8220/18/11/3905/s1, Video S1_Tetra_normal: Normal opening/closing dynamics of the valve leaflets, in tetrapolar impedance measurement configuration. Video S2_Bipolar_normal: Normal opening/closing dynamics of the valve leaflets, in bipolar impedance measurement configuration. Video S3_Tetra_altered: Altered opening/closing dynamics caused by the sponge obstructing the complete valve closure, in tetrapolar impedance measurement configuration.
